# The Nature of Paired Associate Learning Deficits in Chinese Children with Developmental Dyslexia

**DOI:** 10.3390/brainsci13020172

**Published:** 2023-01-19

**Authors:** Ting Yang, Yan Cai, Hong Liu, Xiangping Liu

**Affiliations:** 1 School of Psychology, Beijing Normal University, Beijing 100875, China; 2 Beijing Key Laboratory of Applied Experimental Psychology, School of Psychology, Beijing Normal University, Beijing 100875, China

**Keywords:** dyslexia, paired associate learning, cross-modal, verbal, priming task

## Abstract

Previous studies have found that individuals with dyslexia perform poorly in paired associate learning (PAL) tasks, which were explained by a deficit in cross-modal association or verbal demand in alphabetic language. However, the nature of PAL deficits in non-alphabetic languages remains unclear. In this study, we conducted PAL and priming tasks in visual–visual, visual–verbal, verbal–visual, and verbal–verbal conditions to dissociate the cross-modal and verbal demands in Chinese children with dyslexia. In Experiment 1, children with dyslexia performed worse in verbal-involved PAL (visual–verbal, verbal–visual, and verbal–verbal) than the control children. Experiment 2 revealed that children with dyslexia performed better than the control children in the verbal–visual condition. Our results suggest that children with dyslexia have an intact ability to form cross-modal associations, which also implies that phonological deficits might be the key to PAL deficits in Chinese children with dyslexia.

## 1. Introduction

Developmental dyslexia is characterized by difficulties with accurate/fluent word recognition and poor spelling and decoding abilities, which is a specific learning disability that is neurobiological in origin [1]. The incidence of developmental dyslexia is about 5–15% in different regions [2,3]. A fundamental step in word learning is to create arbitrary associations between orthography and phonology [4], which are usually measured using paired associate learning (PAL). PAL taps the ability to associate one stimulus with another (visual or verbal). If children with dyslexia cannot effectively form an association between visual and verbal items, then word learning would be impaired, further affecting reading performance.

Deficits in visual–verbal PAL have been found in individuals with dyslexia across languages [2,5,6,7,8,9]. Nevertheless, the process of visual–verbal PAL includes at least three components: visual item, verbal item, and the association between the two items [8,9,10]. Deficits in one or more components may lead to the failure of visual–verbal PAL performance, which may further result in dyslexia. This leads to an open question: what causes the deficit in visual–verbal PAL in dyslexia?

Two views explain the deficit in visual–verbal PAL in dyslexia. One view is that there is a deficit of visual–verbal PAL locus in the cross-modal association. Some studies have found that visual–verbal PAL is a unique predictor of reading abilities, even after considering phonological awareness [6,10,11,12] and the ability to remember visual or verbal items [13,14]. One limitation is that taking the single-modal (visual or verbal) processing ability into account statistically is an indirect way to examine the verbal role in the cross-modal task. However, verbal processing in a single modality cannot simply replace phonological processing in the cross-modal association, since cross-modal association requires a particular allocation of cognitive resources in the visual, verbal, and association processes.

Some studies used a more comprehensive PAL task in the visual–visual, visual–verbal, verbal–visual, and verbal–verbal conditions. This provides the opportunity to effectively examine the role of visual, verbal, and cross-modal associations in the process of two-stimulus association. Another view is that verbal deficits mainly cause deficits in visual–verbal PAL with dyslexia. Some studies compared the performance of PAL tasks in visual–visual, visual–verbal, verbal–visual, and verbal–verbal conditions between children with dyslexia with age-matched controls [8,9]. The results revealed that verbal-output PAL performance (visual–verbal and verbal–verbal) was significantly correlated with reading ability. Children with dyslexia performed worse in those two conditions than the control group did. Moreover, the study further found that poor readers learned at the same rate as those in the control group in verbal–visual PAL but showed impairments in visual–verbal PAL using the same paired associate stimuli [15]. This suggests that PAL deficits reflect impairment in the phonological system of visual–verbal PAL.

However, related studies using the same method have only been conducted for alphabetic scripts [8,9]. Less is known about non-alphabetic languages, such as Chinese, with higher visual complexity and less transparency in orthography–phonology mapping. Moreover, there are many homophones in Mandarin Chinese. This means that more than two characters share the same syllable, necessitating a greater need for the orthographic–phonological association in Chinese children. In Chinese studies, only one or two PAL conditions (visual–visual, visual–verbal) were used to measure the performance of Chinese children’s reading abilities. It was found that visual–verbal PAL was significantly related to word recognition, and children with dyslexia showed worse performance than the control group in the visual–verbal condition than in the visual–visual condition [6,12,16,17]. However, only comparing the two conditions is not enough to fully explore the nature of visual–verbal PAL deficits in dyslexia. The visual–visual condition is the association of two visual items in the same modal. By contrast, the visual–verbal condition represents the association between the two models and the addition of a phonological process. Comparing these two conditions makes it impossible to distinguish the visual–verbal impairment caused by cross-modal or phonological deficits. Therefore, adopting PAL tasks across four conditions would contribute to a comprehensive understanding of the PAL deficits in Chinese children with dyslexia.

In addition, a priming test was added to investigate the ability of associating the two items after the PAL task was completed. The priming task is an implicit test to measure association ability, requiring the subject to judge whether the two stimuli are matched. It is a recognition task that does not require further output processes of the verbal or visual stimuli. If children with dyslexia performed worse than the control groups in the priming task, it could be considered that children with dyslexia have a deficit in the association. This task is an effective complement to PAL under all four conditions.

This study aimed to investigate the nature of paired associate learning deficits in Chinese children with dyslexia. Experiment 1 focused on the PAL task performance of Chinese children with dyslexia and control children in the visual–visual, visual–verbal, verbal–visual, and verbal–verbal conditions. Experiment 2 used the priming task across these four conditions to further examine whether the deficit in cross-modal association caused a deficit in PAL. Due to the high demand for orthography–phonology association and the simplicity of syllables in Chinese, we hypothesized that children with dyslexia would perform worse than the control children in the visual–verbal and verbal–visual conditions in both PAL and priming tasks rather than in the visual–visual and verbal–verbal conditions.

## 2. Experiment 1

Performance in PAL depends on three components: visual item, verbal item, and the association between the two items [10]. This study examined the nature of PAL deficits in Chinese children with dyslexia. To do so, we used four PAL conditions (visual–visual, visual–verbal, verbal–visual, and verbal–verbal) to investigate the differences in performance between Chinese children with dyslexia and the control children.

### 2.1. Method

#### Participants

A total of 62 children were recruited from a primary school with 517 students in grades three to five in Beijing, China. The screening for developmental dyslexia in Chinese children meets the following criteria: (1) score in the Character Recognition Measure and Assessment Scale for Primary School Children [18] being at least 1.5 SD below their corresponding grades; (2) score in the Raven’s Standard Progressive Matrices [19] being at least above 45% to exclude those with low IQ. In addition, the ADHD Corners Teacher Rating Scale was filled out by teachers to exclude children with ADHD. All children were native Mandarin speakers with normal hearing and normal or corrected-normal vision without neurological abnormalities. In addition, phonological awareness was measured in all children. There were 31 Chinese children with developmental dyslexia and 31 age-matched normal children in the final samples. The two groups differed significantly in their character recognition, *F* (1, 60) = 82.509, *p* < 0.05, phonological awareness, *F* (1, 60) = 11.715, *p* < 0.05, but not on Raven’s Standard Progressive Matrices, *F* (1, 60) = 2.886, *p* > 0.05, or age, *F* (1, 60) = 1.421, *p* > 0.05 (Table 1). The experiments were approved by the Academic Committee of the School of Psychology at Beijing Normal University, China.

### 2.2. Stimuli and Measures

#### 2.2.1. Stimuli

**Figure 1:**
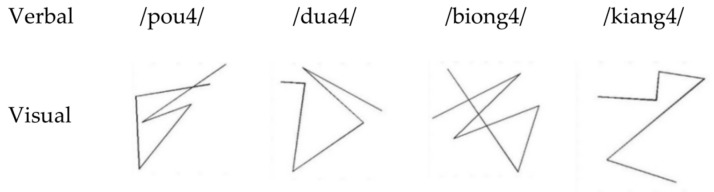
Examples of visual and verbal stimuli in the paired associate learning task.

Visual stimuli: Sixteen abstract shapes were generated using MATLAB software. They were composed of five-line segments of 142 × 142 pixels. The shapes in black lines on a white background were randomly divided across four PAL tasks (Figure 1).

Verbal stimuli: The verbal stimuli in this experiment were pseudowords that could be pronounced but did not exist in Chinese (e.g., /pou4/, 4 represents the fourth tone in Chinese). Since Duff’s study found that familiarity with word pronunciation would promote word reading [20]. The onset and rhyme of each pseudoword were different from others among the four verbal stimuli in each PAL condition, but the tone in all verbal stimuli was the same. The speech materials recorded by a professional male were finally cut into 500 ms lengths and randomly matched with the corresponding material in four conditions.

#### 2.2.2. Measures

Character Recognition: The children were instructed to create words based on the given characters. The examination consisted of 210 words divided into 10 sections, each arranged in the ascending order of difficulty. One point was awarded for each correct answer. The scores of the ten groups were weighted according to the degree of difficulty for each group to achieve the total score.

Raven’s Standard Progressive Matrices: The test was used to evaluate children’s non-verbal intelligence. The test consists of 60 questions divided into five groups of increasing difficulty. Children were asked to complete the target pattern by selecting the most appropriate option of the 6-8 alternative patterns by reasoning. One point was awarded for each correct answer for a total score of 60.

Phonological awareness: The participant was instructed to delete the initial, middle, or final sound from the presented syllable and to articulate the remainder. Each section contained 3 practice trials and 8–10 test items. One correct response was worth 1 point, with a maximum score of 28 possible points.

### 2.3. Procedure

The children were individually tested in a quiet room. Children completed one condition of the PAL task per week separately to minimize interference across the four task conditions (visual–visual, visual–verbal, verbal–visual, and verbal–verbal). The order of the four PAL tasks was counterbalanced among children. The experiments were run in E-prime 1.1 on a 14-inch laptop.

According to the procedure of previous studies [8,9], four stimulus–response pairs were presented in each PAL condition with two blocks (presentation block and test block). In the presentation block, the children were instructed to repeat each pseudoword aloud or draw the abstract shape in a booklet according to the response item. When referring to the previous response, the booklet was set up to let the children draw one shape on each page. If the children produced the wrong item, the experimenter would correct it immediately and ask them to repeat the right one. Then, four stimulus–response pairs were presented individually. The children were asked to remember the mapping of their stimulus–response. After the four pairs finished the presentation, a stimulus item was presented on the screen, and the children were asked to produce a response that matched this stimulus.

The test block contained six trials with four stimulus–response pairs. In each trial, the stimulus item was presented on the screen, and the children were asked to produce a paired response item (speak out the verbal item or draw the visual item). The correct answer was presented, regardless of whether the children gave the correct answer. In the pilot study, we found the task was difficult to have a floor effect, a result consistent with the previous study [9]. Therefore, in this experiment, the children were asked to obtain at least two points during the first trial of the testing block. Otherwise, the presentation block would be repeated until the children received two points.

### 2.4. Results

The experiment included three dependent variables. The score in the last trial was calculated from the number of correct answers in the sixth trial of the test block, as the index of the final learning effect. The number of practices in a presentation block was calculated from the time of practice till the children obtained two correct answers for the first time in four stimulus–response pairs, as the index of the early learning effect. The total score in the six learning trials was calculated from the total number of correct answers in the test block, as the index of the late learning effect.

One-way ANOVA was used to analyze the learning effect between dyslexic and control groups in the four PAL task conditions for three dependent variables.

**Figure 2:**
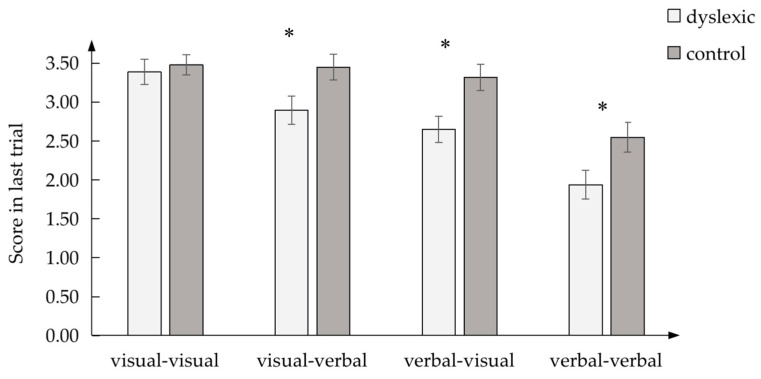
Final learning effect in four PAL conditions between dyslexic and control groups; * *p* < 0.05.

In the final learning effect (Figure 2), there was a significant difference between the dyslexic and control groups in the following realms: visual–verbal, *F* (1, 60) = 4.960, *p* < 0.05, *η_p_*^2^ = 0.076; verbal–verbal, *F* (1, 60) = 5.330, *p* < 0.05, *η_p_*^2^ = 0.082; and verbal–visual, *F* (1, 60) = 7.922, *p* < 0.05, *η_p_*^2^ = 0.117. However, there was no difference in the visual–visual condition, *F* (1, 60) = 0.212, *p* > 0.05, *η_p_*^2^ = 0.004. Thus, children with dyslexia only learned less than the control children in verbal-involved conditions (visual–verbal, verbal–visual, and verbal–verbal) and not in the visual–visual conditions.

**Figure 3:**
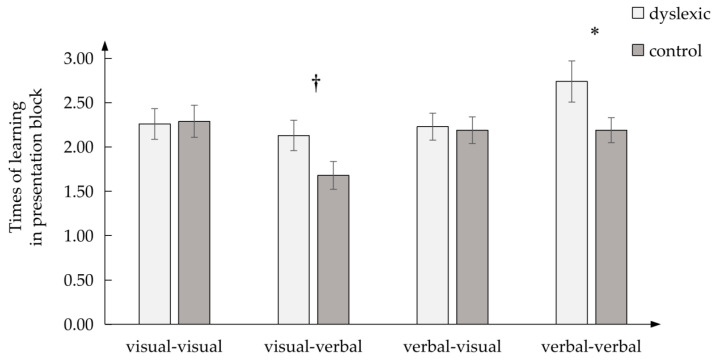
The early learning effect in four PAL conditions between dyslexic and control groups; * *p* < 0.05; 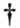 0.05 < *p* < 0.10.

In the early learning effect (Figure 3), there was a marginally significant difference in the visual–verbal condition, *F* (1, 60) = 3.774, *p* = 0.057, *η_p_*^2^ = 0.059, and a significant difference in the verbal–verbal condition, *F* (1, 60) = 4.067, *p* < 0.05, *η_p_*^2^ = 0.063, between the dyslexic and control groups, but there was no difference in the verbal–visual, *F* (1, 60) = 0.023, *p* > 0.05, *η_p_*^2^ = 0.000, or the visual–visual condition, *F* (1, 60) = 0.017, *p* > 0.05, *η_p_*^2^=0.000.

**Figure 4:**
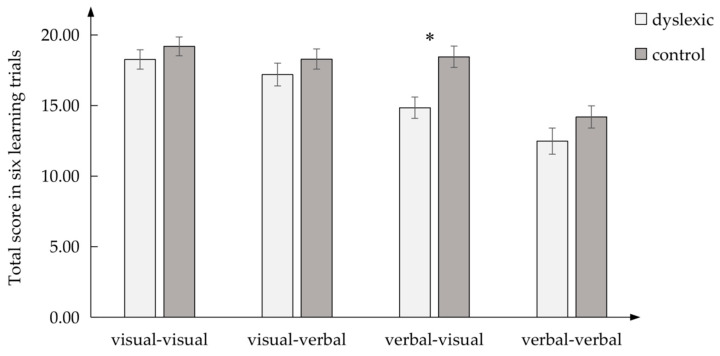
The late learning effect in four PAL conditions between dyslexic and control groups; * *p* < 0.05.

In the late learning effect (Figure 4), there were significant differences between the dyslexic and control groups in the verbal–visual condition, *F* (1, 60) = 11.368, *p* < 0.05, *η_p_*^2^ = 0.159, but there was no difference in the visual–verbal, *F* (1, 60) = 1.035, *p* > 0.05, *η_p_*^2^ = 0.017; verbal–verbal, *F* (1, 60) = 1.963, *p* > 0.05, *η_p_*^2^ = 0.032; and visual–visual condition, *F* (1, 60) = 0.959, *p* > 0.05, *η_p_*^2^ = 0.016.

In addition, children with dyslexia demonstrated equal performance as control children in the visual–visual condition in every stage of learning, all *ps* > 0.05.

### 2.5. Discussion

Experiment 1 aimed to determine the nature of PAL deficits in Chinese children with dyslexia for PAL tasks across visual–visual, visual–verbal, verbal–visual, and verbal–verbal conditions. The results showed that Chinese children with dyslexia performed worse than control children in verbal-involved conditions (visual–verbal, verbal–verbal, and verbal–visual). These results suggest that verbal deficits might be critical for PAL deficits in Chinese children with dyslexia.

Children with dyslexia performed worse in visual–verbal PAL but were not as impaired in visual–visual PAL as the control children, which is consistent with the findings of previous studies [6,7,21]. The addition of verbal–visual and verbal–verbal conditions to our design made it possible to determine the role of verbal demand and cross-modal association in visual–verbal PAL deficits in dyslexia. The results showed that children with dyslexia were impaired in verbal–visual and verbal–verbal PAL, which is inconsistent with the results of a previous study. The study found that children with dyslexia who are native English speakers have a deficit in verbal–verbal PAL but not in verbal–visual PAL [9]. There are two possible reasons for the inconsistent results. One reason might be the procedure difference. In our study, children needed to obtain two points in four pairs in the early learning stage. We found that children with dyslexia performed well compared with the control groups in the early learning stage but began to show impairment in the later stage under the verbal–visual condition. However, Litt’s study only focused on the six types of learning PAL tasks in the verbal–visual condition, which is in the relatively early and middle learning stage [9]. Thus, children with dyslexia may not present a deficit at this stage. Another reason may be the characteristics of the Chinese script. Unlike the simple symbols used in previous studies [6,8,9], the irregular shapes of the random combination used in this study are visually complex and closer to the difficulty of visual processing in Chinese characters. Therefore, it might be difficult for Chinese children with dyslexia to recall visual items in the verbal–visual condition. This confirms that they have a deficit in dictation, a classic characteristic of the subtype of children with dyslexia [22].

In addition, the results showed that children with dyslexia had different deficit patterns across the different learning stages of PAL conditions. In the visual–verbal and verbal–verbal conditions, children with dyslexia learned less in the early stage, but had a learning rate similar to that of the control group in the late stage. In contrast, in verbal–visual conditions, children with dyslexia had a similar learning rate in the early stage with control groups, but improved less in the late stage. This implies that further research is required to consider the learning stage.

However, as the study mentioned, PAL deficits might be accounted for by verbal demands or cross-modal association [9]. For deficits found in the visual–verbal and verbal–visual conditions, this experiment remains an unsolved problem for whether Chinese children with dyslexia have cross-modal deficits simultaneously. To dissociate the verbal demand and cross-modal association in PAL deficits, in Experiment 2, we used a priming task to focus on the association process across the four conditions to determine whether the association could be efficiently formed in children with dyslexia.

## 3. Experiment 2

In Experiment 2, we used the priming task to investigate whether the association between two items could be effectively formed in Chinese children with dyslexia under four conditions (visual–visual, visual–verbal, verbal–visual, and verbal–verbal). Priming is the phenomenon in which a previously presented stimulus (priming item) influences the response to a subsequent stimulus (target item), resulting in a preference manifested as faster response speed, greater accuracy, etc. If the priming stimuli are matched with the following target item that children have learned in the PAL task, the response accuracy will increase in the dyslexic and control groups; if not matched, the accuracy will decrease.

### 3.1. Method

#### 3.1.1. Participants

The children who participated in Experiment 2 were the same as those in Experiment 1.

#### 3.1.2. Stimuli

This experiment included the materials used in Experiment 1, but eight visual and eight verbal response items were added as interference items. Therefore, 24 visual and 24 verbal stimuli were used.

#### 3.1.3. Procedure

There were four priming tasks (visual–visual, visual–verbal, verbal–visual, and verbal–verbal). All the children completed the priming task immediately after the corresponding PAL task, ensuring they learned the association between two items. Each task included 48 trials.

**Figure 5:**
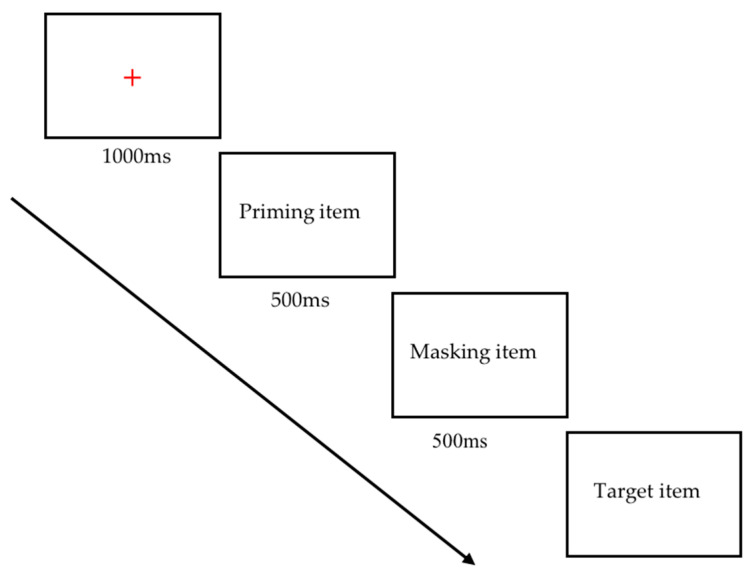
A trial in the priming task.

In each trial (Figure 5), the fixation point was first presented at 1000 ms to remind the child to maintain their attention. The priming item was shown at 500 ms. The priming item was the first stimulus in different conditions (visual–visual, visual–verbal, verbal–visual, and verbal–verbal). If the priming item was a visual stimulus, then the following masking item was a scrambled line. If the priming item was a verbal stimulus, the next masking item was a 1000 HZ pure tone of 500 ms. After the masked item was presented, the target item was shown. There were two conditions for target items: either the priming item and target item were matched in the preceding PAL task, or they were not —the target item was learned but not matched with the priming item, or the target item had not been learned before. Children were asked whether the target item had been learned in the previous PAL task. The proportion of correct responses across all trials was recorded as the response accuracy in each condition.

### 3.2. Results

**Figure 6:**
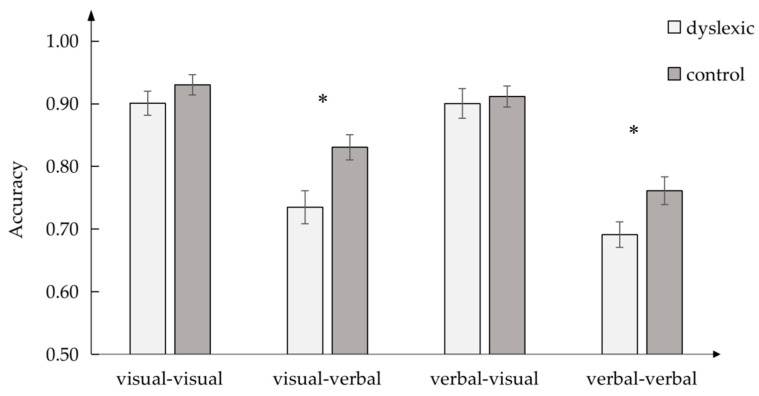
Accuracy in priming task in four PAL conditions between dyslexic and control groups; * *p* < 0.05.

Accuracy was calculated between the dyslexic and control groups (Figure 6). One-way ANOVA was used to analyze the differences between the dyslexic and control groups in the four conditions separately.

To check the manipulation effect of PAL, a t-test with a percentage of 50% showed that the dyslexic and control groups performed better than the random probability, all *ps* < 0.05. The priming task effectively measured the learning effect of two-item mapping in PAL tasks.

Significant differences were observed between the dyslexic and control groups in the visual–verbal condition, *F* (1, 60) = 8.303, *p* < 0.05, *η_p_*^2^ = 0.122, and verbal–verbal PAL, *F* (1, 60) = 5.400, *p* < 0.05, *η_p_*^2^ = 0.083. However, there was no difference in performance between the two groups in the visual–visual condition, *F* (1, 60) = 1.388, *p* > 0.05, *η_p_*^2^ = 0.023, and verbal–visual condition, *F* (1, 60) = 0.157, *p* > 0.05, *η_p_*^2^ = 0.003.

### 3.3. Discussion

In Experiment 2, a priming task was used to examine whether deficits in PAL were simultaneously caused by cross-modal associations. If a cross-modal deficit exists, Chinese children with dyslexia will perform worse than control groups in visual–verbal and verbal–visual conditions. This result demonstrated that children with dyslexia did not differ from the control group in the verbal–visual condition. Thus, children with dyslexia have an intact ability to form cross-modal associations. This illustrates that the deficits in PAL in Chinese children with dyslexia did not result from cross-modal deficits.

This experiment found that the cross-modal ability of children with dyslexia was intact, which is consistent with the findings of previous studies in alphabetic scripts [7,9]. No difference was found in the verbal–visual condition using the recognition task [7] and the recall task [9]. This study extends the previous research into the non-alphabetic script, which further suggests that the nature of the PAL deficit in Chinese children with dyslexia rules out the cross-modal hypothesis.

Compared to the control group, this study also found that children with dyslexia were impaired in the visual–verbal and verbal–verbal conditions, indicating that the association between the two items was not well-formed. This may be due to impairment in verbal input processing as children with dyslexia have their cross-modal association abilities intact. This result rules out the hypothesis of a verbal output deficit because the priming paradigm only focuses on examining the effects of cross-modal association without the output process. However, why do children with dyslexia demonstrate an association deficit in the visual–verbal condition but not in the verbal–visual condition? This may be due to the different requirements of the phonological representation quality across these two conditions. In the visual–verbal condition, all phonological features must be remembered, and no single phoneme can be wrong when obtaining scores. However, in the verbal–visual condition, children with dyslexia can learn verbal stimuli by only memorizing parts of the features of the verbal stimuli rather than the full features—the four nonwords are maximally different in phonological features—to complete the verbal–visual PAL task [9,15]. Therefore, it is speculated that there is a phonological deficit in the visual–verbal condition, which leads to dyslexia and requires more cognitive resources to process verbal features, further affecting the subsequent cross-modal association of visual and verbal information.

## 4. General Discussion

Our study explores the nature of PAL deficits in Chinese children with dyslexia using four conditions of the PAL and priming tasks. Experiment 1 assessed the performance of the dyslexic and normal groups across the four conditions of the PAL tasks and found that children with dyslexia only have deficits in the verbal-involved condition. In Experiment 2, we used the priming task after the PAL task was completed across all four conditions and found that children with dyslexia performed as well as the control group in the verbal–visual condition, suggesting an intact ability of cross-modal association. Contrary to our hypothesis, this study implies that deficits in PAL might be mainly caused by verbal deficits in Chinese children with dyslexia rather than cross-modal associations.

The most important finding is that verbal demand plays an especially crucial role in PAL deficits rather than cross-modal association, supporting the verbal account in PAL deficits for Chinese dyslexia, similar to studies in alphabetic language. Litt found that the deficit in PAL with dyslexia in English is mainly caused by the verbal demand for the deficit in verbal-output PAL (visual–verbal and verbal–verbal) [9]. Moreover, it was found that the difference fully accounted for phonological form learning in visual–verbal PAL deficits. Aravena further supported that phonological deficit damaged the following letter–sound integration [2]. The error-type analysis found that individuals with dyslexia exhibit more phonological errors than association errors in paired associate learning tasks [7,15,23]. Our result could be explained by the phonological deficit that children with dyslexia might have had causing ineffective phonological representatives in the encoding stage before the association. Compared with typical readers, the established phonological representation of dyslexia has some specific characteristics: the representation is fuzzy, the representation’s granularity is large, and the categorization degree is not high, which causes the preservation of a large amount of redundant phonological variant information in processing [24,25]. As for the hypothesis of phonological deficits, studies have revealed that children with Chinese dyslexia have deficits in phonological awareness and that phonological awareness can be used to distinguish children at risk of dyslexia from normal children [26]. The studies of different types of phonological awareness tasks have also found that phonological awareness can effectively predict the ability to recognize Chinese characters, especially in preschool or early primary school [27,28,29,30,31,32]. The study also found that children with dyslexia performed equally well as the control groups in the visual–visual condition. This is consistent with studies in alphabetic language, suggesting that Chinese children with dyslexia are good at visual processing. In some studies, the visual item revealed a semantic cue that might help them remember the item. For example, one visual item is a novel animal picture constructed by combining a familiar animal with a new feature [6]; animal pictures such as cats, dogs, and fish [7]; or alien pictures [14]. These meaningful visual stimuli might have been confounding factors in the results. Our study expands the visual item into abstract and meaningless shapes and finds that children with dyslexia can effectively process visual–visual pairs in Chinese dyslexia. This suggests that children with dyslexia have intact visual ability, which implies that visual demand is not a critical factor in PAL deficits.

This study expands on the nature of deficits in PAL in Chinese dyslexia, enriching the understanding of PAL deficits in non-transparent languages. In addition, this study revealed that verbal demand is significant when considering the deficits of PAL, which is conducive to providing targeted teaching and intervention, such as reading aloud to strengthen phonological processing, rather than visual processing or cross-modal association.

This study had some limitations. First, the span of memory in PAL needs to be considered. In this study, we used four pairs of stimuli responses, which is relatively lower than in previous studies, to minimize the floor effect. However, the span of PAL in dyslexia is less than three pairs [33], which might influence children with dyslexia performance. Second, we only focused on the accuracy of the PAL tasks in this study. However, learning speed, which is also an important index for judging learning ability, was not considered; future studies should consider learning speed when examining the causes of PAL deficits in children with dyslexia.

## 5. Conclusions

This study examines the nature of PAL deficits in Chinese children with dyslexia. The results demonstrated that phonological deficits could explain PAL deficits rather than cross-modal associations in dyslexia. This study provides effective guidance, emphasizing phonological practices for future interventions in Chinese dyslexia.

## Figures and Tables

**Table 1:** Descriptive characteristics of Chinese dyslexic and control groups.

Measures	Dyslexic Group (*n* = 31)		Control Group (*n* = 31)	
	*M*	*SD*	*M*	*SD*
Age (years)	10.01	0.972	10.30	0.984
Character recognition	1796.67	423.630	2684.23	341.339
Raven’s Standard Progressive Matrices (%)	72.98	14.326	79.30	14.959
Phonological awareness	19.06	4.632	22.32	2.574

## Data Availability

All data related to the research are presented in the article.
